# Endovascular treatment of a pancreatic pseudoaneurysm associated with massive pancreatic arteriovenous malformation using flow modification technique

**DOI:** 10.1093/bjrcr/uaag005

**Published:** 2026-02-17

**Authors:** Kohei Hamamoto, Ryoma Kobayashi, Emiko Chiba, Naoki Kunitomo, Soichiro Kojima, Hiroyuki Fujii, Mitsuru Matsuki, Kazuma Rifu, Homare Ito, Mineyuki Tojo, Harushi Mori

**Affiliations:** Department of Radiology, Jichi Medical University, Shimotsuke-city, Tochigi 329-0498, Japan; Department of Radiology, Jichi Medical University, Shimotsuke-city, Tochigi 329-0498, Japan; Department of Radiology, Jichi Medical University, Shimotsuke-city, Tochigi 329-0498, Japan; Department of Radiology, Jichi Medical University, Shimotsuke-city, Tochigi 329-0498, Japan; Department of Radiology, Yuai Memorial Hospital, Koga-city, Ibaraki 306-0232, Japan; Department of Radiology, Jichi Medical University, Shimotsuke-city, Tochigi 329-0498, Japan; Department of Radiology, Jichi Medical University, Shimotsuke-city, Tochigi 329-0498, Japan; Department of Radiology, Jichi Medical University, Shimotsuke-city, Tochigi 329-0498, Japan; Division of Gastrointestinal, General and Transplant Surgery, Department of Surgery, Jichi Medical University, Shimotsuke-city, Tochigi 329-0498, Japan; Division of Gastrointestinal, General and Transplant Surgery, Department of Surgery, Jichi Medical University, Shimotsuke-city, Tochigi 329-0498, Japan; Division of Gastrointestinal, General and Transplant Surgery, Department of Surgery, Jichi Medical University, Shimotsuke-city, Tochigi 329-0498, Japan; Department of Radiology, Jichi Medical University, Shimotsuke-city, Tochigi 329-0498, Japan

**Keywords:** pancreatic arteriovenous malformation, pseudoaneurysm, transcatheter arterial embolisation

## Abstract

Pancreatic arteriovenous malformation (PAVM) is a rare vascular anomaly of the gastrointestinal tract, and the coexistence of a pseudoaneurysm is exceptionally uncommon. This report details a case of pseudoaneurysm associated with a massive PAVM that was successfully managed with endovascular therapy. A 67-year-old female patient with a massive PAVM presented with acute back pain. Contrast-enhanced computed tomography revealed a pseudoaneurysm in the pancreatic head accompanied by a surrounding haematoma. Transcatheter arterial embolisation (TAE) was selected as the minimally invasive treatment and the pseudoaneurysm was visualised on angiography. However, identifying the culprit artery was challenging due to the extensive vascular network of the PAVM. Following modification of the local haemodynamics using coil embolisation, the culprit artery became detectable, allowing successful embolisation of the pseudoaneurysm using N-butyl cyanoacrylate (NBCA). This case report highlights the clinical utility of NBCA embolisation combined with flow modification for managing a pseudoaneurysm associated with a massive PAVM, which represents an exceptionally rare vascular anomaly.

## Introduction

Pancreatic arteriovenous malformations (PAVMs) are rare vascular anomalies with an incidence of approximately 0.9%.^1^ Nearly 90% of cases are considered congenital, typically resulting from abnormal development of the arteriovenous plexus during embryogenesis. Other causes of acquired pancreatic AVMs include trauma, pancreatitis, iatrogenic factors, and various other conditions.^2^ Among these rare conditions, cases further complicated by pancreatic pseudoaneurysms are exceptionally rare, with only a few cases reported to date.^3^

This report presents a rare case of impending rupture of a pancreatic pseudoaneurysm that developed during the follow-up of a massive PAVM. The patient was successfully managed using selective embolisation combined with flow modification.

### Case presentation and imaging findings

**Figure 1.**
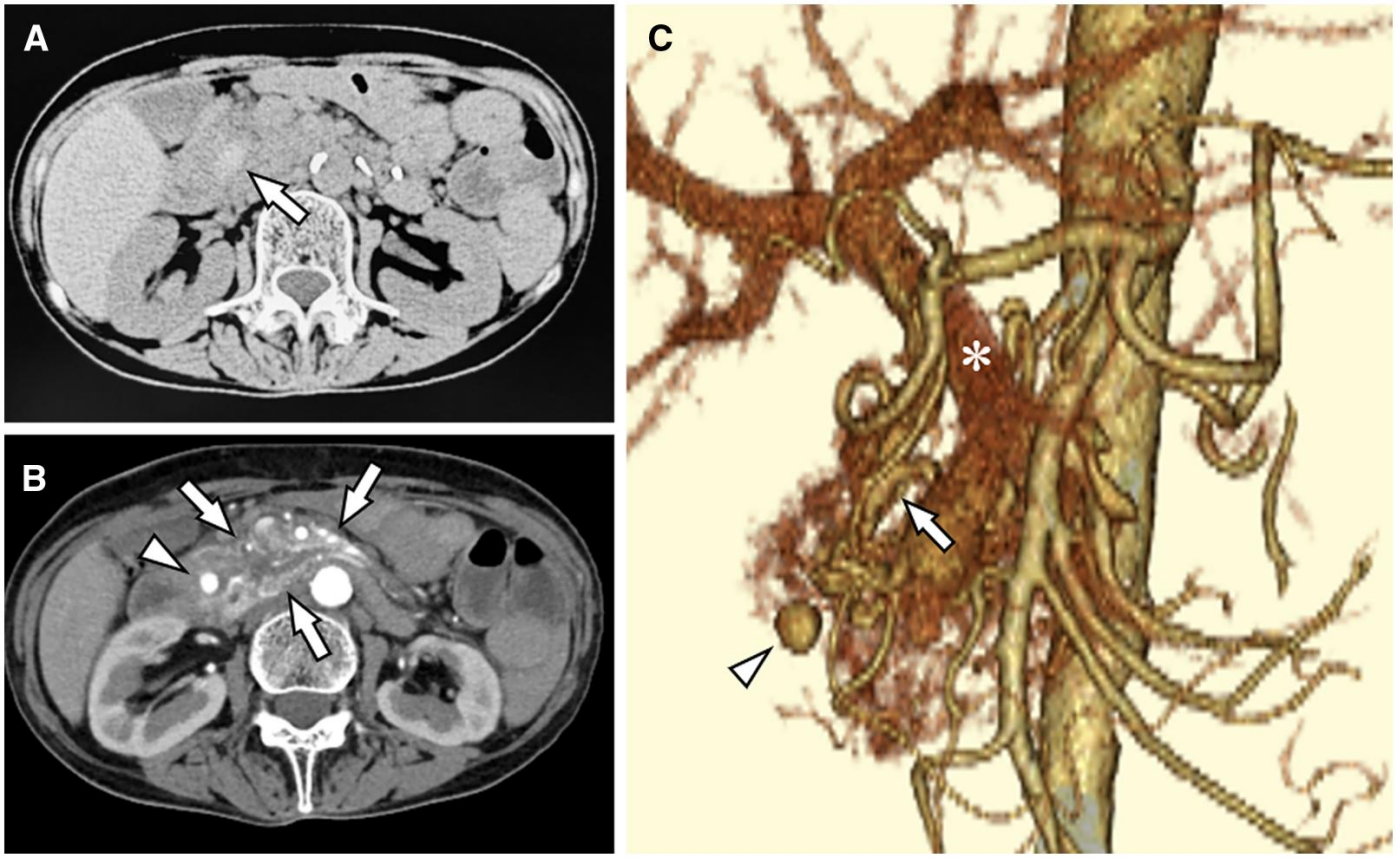
Computed tomography (CT) on admission. (A) Non-contrast-enhanced CT shows a hyperdense area in the pancreatic head, suggestive of a haematoma (arrow). (B) Contrast-enhanced CT in the arterial phase reveals a pseudoaneurysm in the pancreatic head (arrowhead) and vascular proliferation consistent with a pancreatic arteriovenous malformation (PAVM) extending from the head to the body of the pancreas (arrows). (C) Volume-rendered image in the arterial phase (coronal view) shows vascular proliferation from the pancreatic head to the body, consistent with a PAVM and a pseudoaneurysm in the pancreatic head (arrowhead). The inferior pancreaticoduodenal and portal veins are also visualised (arrow and asterisk, respectively), suggesting a diagnosis of high-flow PAVM.

A 67-year-old old female diagnosed with a PAVM predominantly affecting the head and body of the pancreas, first identified 10 years prior. As there were no associated complications, she was managed with regular follow-up, although the lesion showed a gradual tendency to increase in size. Her medical history included refractory sigmoid diverticulitis, sarcoidosis with cardiac involvement, and early-stage cervical cancer, which was treated with conization and radiation therapy. Three months prior to presentation, she had undergone a sigmoidectomy for refractory sigmoid diverticulitis. The patient’s post-operative course was uneventful, and she was followed up on an outpatient basis. However, she presented to the emergency department with the chief complaint of abdominal pain. Laboratory tests revealed a haemoglobin level of 8.6 g/dL, which had decreased from 9.6 g/dL 5 days prior. Serum amylase and lipase levels were 133 and 92 U/L, respectively. Contrast-enhanced computed tomography (CT) revealed an 8-mm aneurysmal lesion in the pancreatic head, accompanied by surrounding high-density areas on non-contrast CT, suggestive of a haematoma (Figure 1). This finding was not observed on contrast-enhanced CT performed 3 months earlier. There were no significant changes in the pre-existing PAVM. Multiplanar reconstruction images from various angles revealed no evidence of celiac artery stenosis. A diagnosis of impending rupture of a pancreatic pseudoaneurysm was made, which necessitated prompt management. The culprit vessel was presumed to be the posterior superior pancreaticoduodenal artery, branching from the gastroduodenal artery. A multidisciplinary discussion involving the departments of gastrointestinal surgery, gastroenterology, and radiology concluded that transcatheter arterial embolisation (TAE) is the first line minimally invasive management. The patient and her family consented to participate in this management plan.

## Treatment

**Figure 2.**
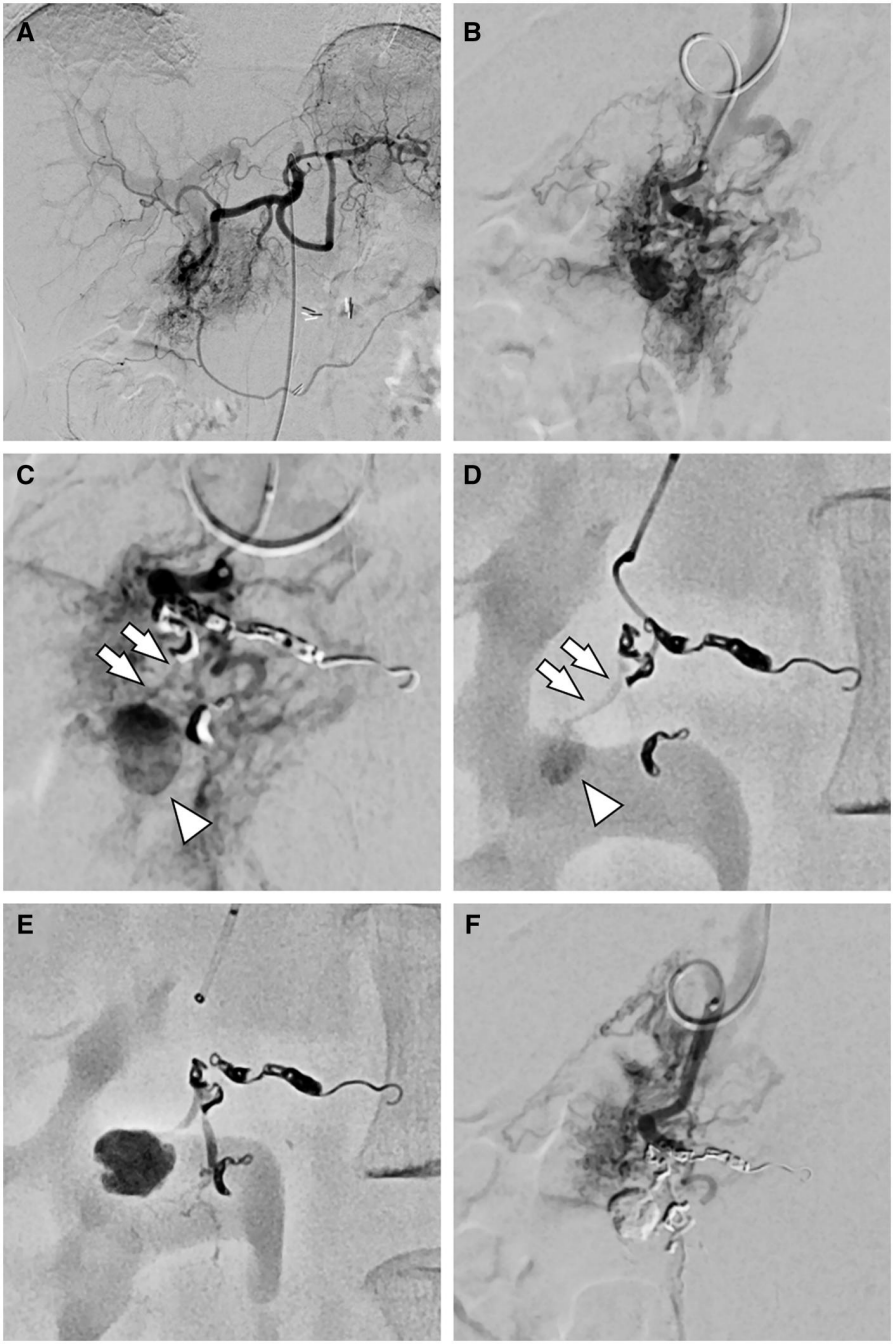
Transcatheter arterial embolization (TAE). (A) Celiac artery angiography. A massive PAVM is observed in the region of the gastroduodenal artery and the dorsal pancreatic artery. (B) Gastroduodenal artery angiography shows opacification of the pseudoaneurysm (arrow), but identification of the responsible feeding artery is difficult due to the steal phenomenon caused by the PAVM. (C) After flow modification using selective coil embolization of the branches, the feeding artery connected to the pseudoaneurysm becomes clearly visible (arrow). (D) Selective angiography of the culprit artery. Arrowhead indicates the pseudoaneurysm. (E) Fluoroscopic image after injection of a mixture of N-butyl cyanoacrylate and lipiodol shows selective filling of the pseudoaneurysm and its feeding artery. (F) Post-embolization angiography confirms disappearance of the pseudoaneurysm.

Endovascular treatment was performed using a right femoral artery approach. A 5-Fr shepherd-hook catheter was used for superior mesenteric and coeliac artery angiography, which demonstrated a high-flow and massive PAVM consistent with the Cho-Do classification Type 3a+b extending from the pancreatic head to the body (Figure 2A). In the delayed phase, a pseudoaneurysm was identified within the pancreatic head of the PAVM. Celiac artery angiography demonstrated preserved antegrade flow in the pancreaticoduodenal artery territory. A triple coaxial system was constructed using a 1.9-Fr microcatheter (Carnelian MARVEL; Tokai Medical Products, Aichi, Japan) and a 2.8-Fr high-flow microcatheter (Carry LEON; UTM Co., Aichi, Japan). The posterior superior pancreaticoduodenal artery was selected via the gastroduodenal artery and angiography was performed using multiple projections. However, due to the steal phenomenon caused by the massive PAVM and image degradation from bowel motion, it was difficult to identify the feeding artery of the pseudoaneurysm (Figure 2B). Although multiple branches suspected of being connected to the pseudoaneurysm were catheterised, the culprit vessel could not be clearly identified. Therefore, to reduce the steal phenomenon, flow modification was planned by embolising a part of the PAVM. Two PAVM branches near the pseudoaneurysm were selected, and following confirmation that the pseudoaneurysm was no longer visualised on angiography, they were embolised with four 0.0120 inch-microcoils (one 2 mm × 6 cm, one 1.5 mm × 4 cm, and two 1 mm × 3 cm) (AZUR 3D soft; Terumo Corporation, Tokyo, Japan). The feeding artery of the pseudoaneurysm was clearly delineated (Figure 2C). The culprit vessel was then selectively catheterised (Figure 2D) and embolised with a mixture of 20% N-butyl-2-cyanoacrylate (NBCA) (B. Braun Aesculap Japan Co., Ltd., Tokyo, Japan) and Lipiodol (Guerbet Japan Co., Ltd., Tokyo, Japan) (Figure 2E). Post-embolisation angiography confirmed the complete obliteration of the pseudoaneurysm (Figure 2F). Angiography of the superior mesenteric artery also showed no visualization of the pseudoaneurysm via collateral branches.

## Outcome and follow-up

**Figure 3.**
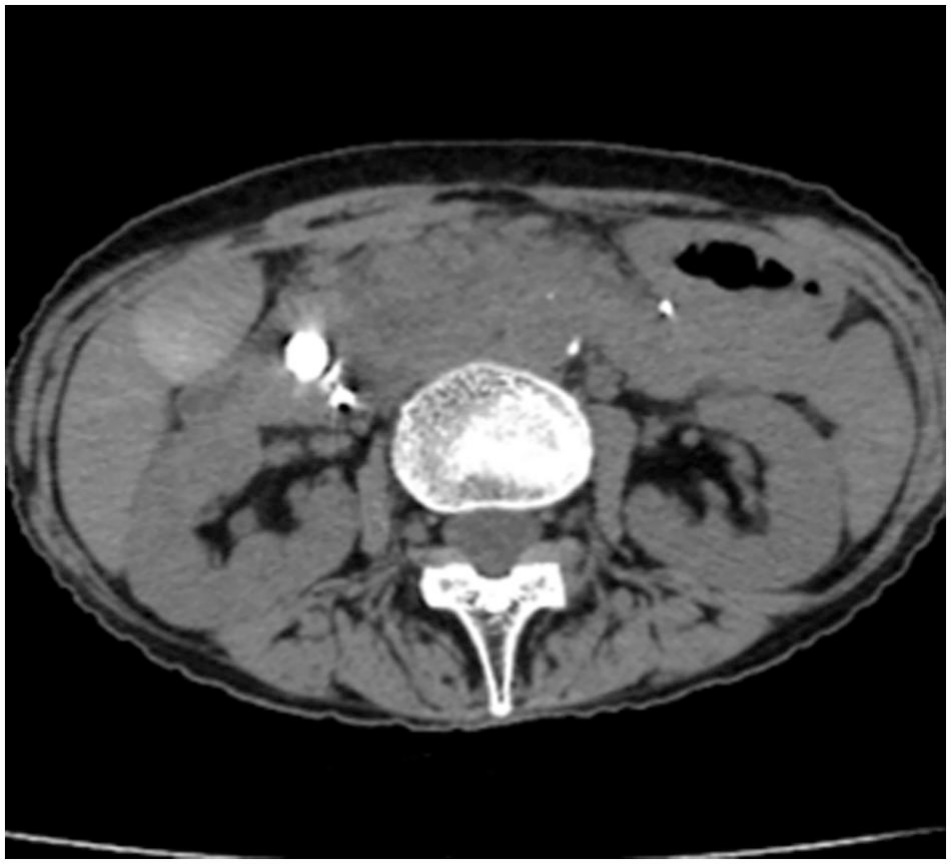
CT five days after TAE. Non-contrast-enhanced CT shows lipiodol deposition corresponding to a pseudoaneurysm in the pancreatic head.

The post-procedural course was uneventful, with no elevation in serum amylase or lipase levels. Follow-up CT performed five days after the procedure showed lipiodol filling within the pseudoaneurysm (Figure 3) without evidence of recanalization, new pseudoaneurysm formation, or bleeding. A small thrombus was observed in the superior mesenteric vein (SMV), and direct oral anticoagulant (DOAC) therapy (apixaban) was initiated. The patient was discharged 12 days post-operatively. Follow-up CT at 3 months demonstrated complete resolution of the thrombus in the SMV. Furthermore, subsequent CT imaging 1 year post-TAE demonstrated no recurrence of the pseudoaneurysm and concurrently showed a slight reduction in the size of the PAVM in the pancreatic head.

## Discussion

PAVM is an uncommon vascular anomaly of the gastrointestinal tract, and the coexistence of a pseudoaneurysm is exceptionally rare. To date, approximately 200 cases of PAVMs have been reported in the literature^2^; however, only one case managed with surgical resection has been reported in Japan.^3^ To the best of our knowledge, this is the first reported case of a pseudoaneurysm arising in the context of a massive PAVM managed with endovascular therapy. While bleeding in PAVM is usually attributed to rupture of the feeding artery or the outflow vein itself,^2^^,^^4^ the present case demonstrated de novo formation of a pseudoaneurysm, as no aneurysmal change was seen on CT performed 3 months earlier. Although the exact mechanism remains elusive, several factors may contribute to the formation of pseudoaneurysms. Localised inflammation or ischaemia related to PAVM may cause vessel wall injury, and the haemodynamic stress of an abnormal high-flow shunt may accelerate this process, similar to the vascular complications seen in pancreatitis. Celiac artery origin stenosis, including median arcuate ligament syndrome, is also known to be associated with true or pseudoaneurysms in the pancreaticoduodenal arcade; however, this was not observed in our case.

Currently, there is no established management strategy for PAVMs with or without pseudoaneurysms; however, surgery, endovascular therapy, and their combinations have been described as potential approaches. Surgical resection has traditionally been the mainstay.^2^ However, endovascular management is increasingly performed as a less-invasive alternative, particularly in patients with bleeding or high surgical risk. According to previous reports, among the 40 reviewed patients who underwent endovascular management, 28 were managed with endovascular therapy alone.^2^ Of these, TAE was successful in approximately 57.7% of cases, whereas rebleeding occurred in approximately 42.3%.^2^^,^^4^ Radiotherapy has been used in limited cases.^5^ Indications for management generally include rupture or impending rupture of the AVM nidus, portal hypertension, and ischaemia caused by the steal phenomenon. In our case, TAE was selected as the first-line management due to the impending rupture of the pseudoaneurysm and the presence of multiple comorbidities. To manage a pseudoaneurysm, complete embolisation of both the inflow and outflow vessels or the pseudoaneurysm itself is required. However, pseudoaneurysm embolisation in the pancreaticoduodenal region is technically demanding due to its complex vascular anatomy, with abundant anastomoses and multiple outflow routes. Furthermore, in cases complicated by PAVM, as in the present case, the haemodynamics become even more complex. In these situations, liquid embolic agents such as NBCA have the advantage of achieving rapid and complete exclusion of the lesion compared with coil embolisation alone. Potential complications of NBCA embolisation include acute pancreatitis, pancreatic necrosis secondary to ischaemia, and portal vein thrombosis, as the embolic material may pass through the AVM during the procedure. In the present case, localised thrombosis of the SMV occurred following embolisation but improved with the administration of DOAC.

In this case, identification of the culprit artery was difficult due to the significant steal phenomenon caused by the high-flow PAVM. By embolising nearby non-essential feeders with coils, the local haemodynamics were modified, thereby allowing clear delineation of the pseudoaneurysm-feeding artery. This approach enables safe and effective targeted NBCA embolisation. Flow modification techniques have been described for endovascular management of AVMs to enhance the selectivity and control of liquid embolic agents.^6^ Since embolisation was performed proximally, collateral perfusion through alternative pathways was preserved, thereby minimising the risk of ischaemic complications. The patient did not experience ischaemic sequelae, and follow-up imaging confirmed stable embolisation. An alternative flow-modification technique involves the use of balloon catheters. Nevertheless, in this case, the extremely small and highly tortuous nature of the culprit artery precluded advancing the balloon catheter to the target site.

In conclusion, this case illustrates the unique presentation of a pseudoaneurysm associated with massive PAVM, which is an exceptionally rare and technically challenging condition. The combination of selective-flow modification and NBCA embolisation provides an effective and minimally-invasive management strategy. This technique may be particularly useful in cases where conventional catheterisation fails to identify the culprit artery due to haemodynamic steal, and may help achieve precise and safe embolisation while reducing the risk of ischaemia.

## Learning points

- Selective embolisation following flow modification represents an effective management strategy for pancreatic pseudoaneurysms associated with high-flow pancreatic arteriovenous malformations (PAVM).
- Coil embolisation of non-essential branches can mitigate the steal phenomenon and delineate the culprit artery, thereby enabling the safe and precise delivery of liquid embolic agents, such as N-butyl cyanoacrylate (NBCA).
- NBCA embolisation offers the advantage of rapid and complete occlusion in complex vascular anatomies, yet necessitates careful monitoring for potential complications such as portal vein thrombosis.

## Ethical approval

Formal ethical approval was not required for this type of study.
